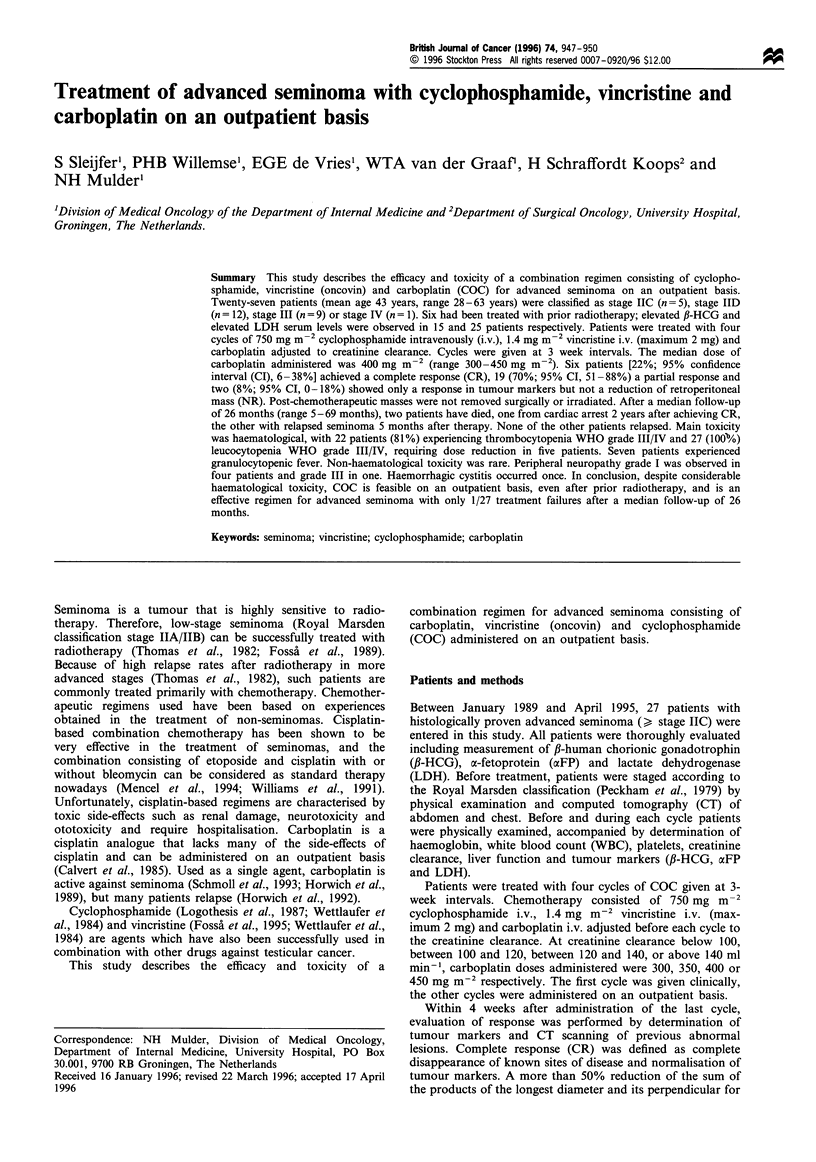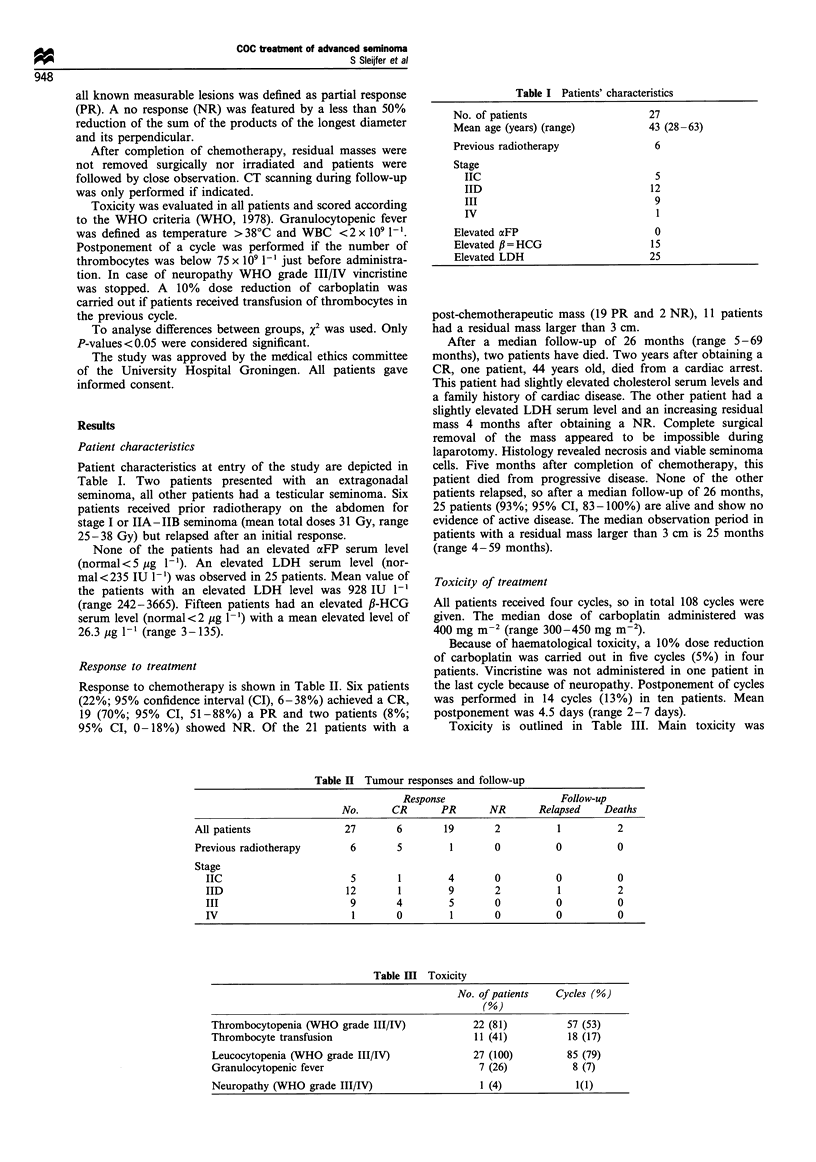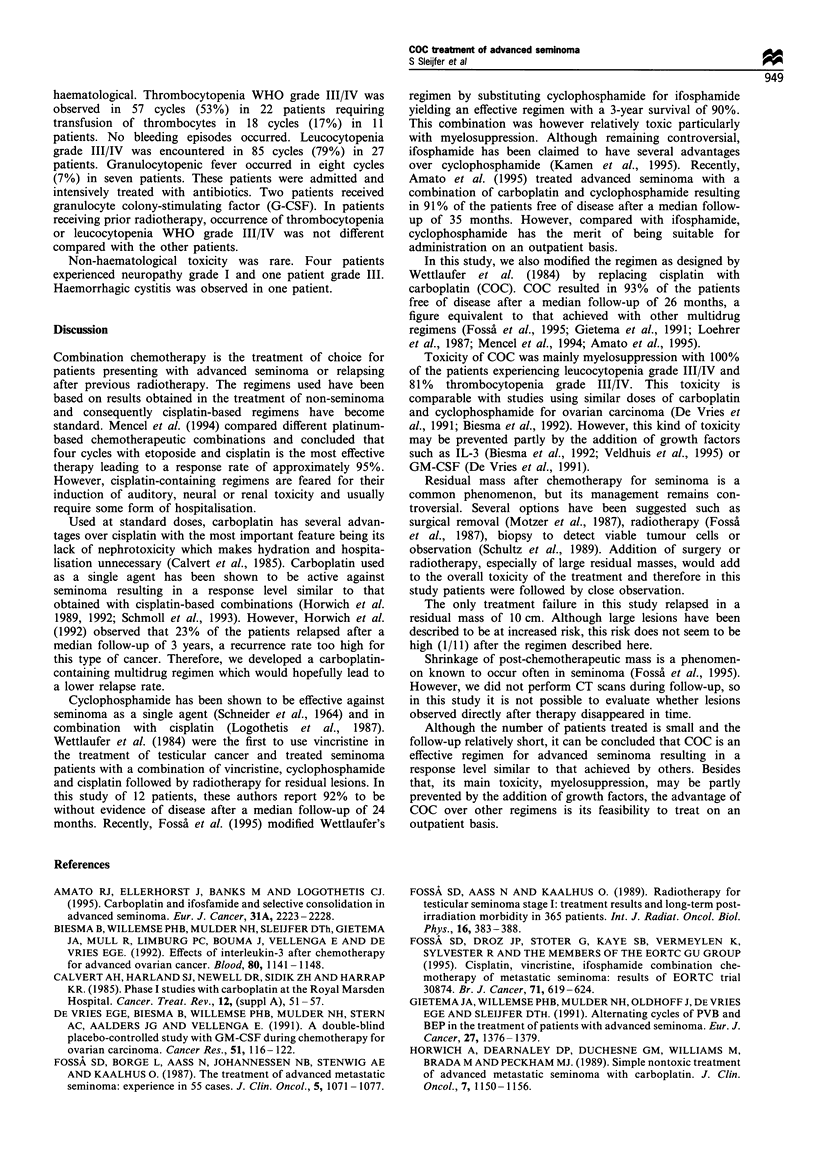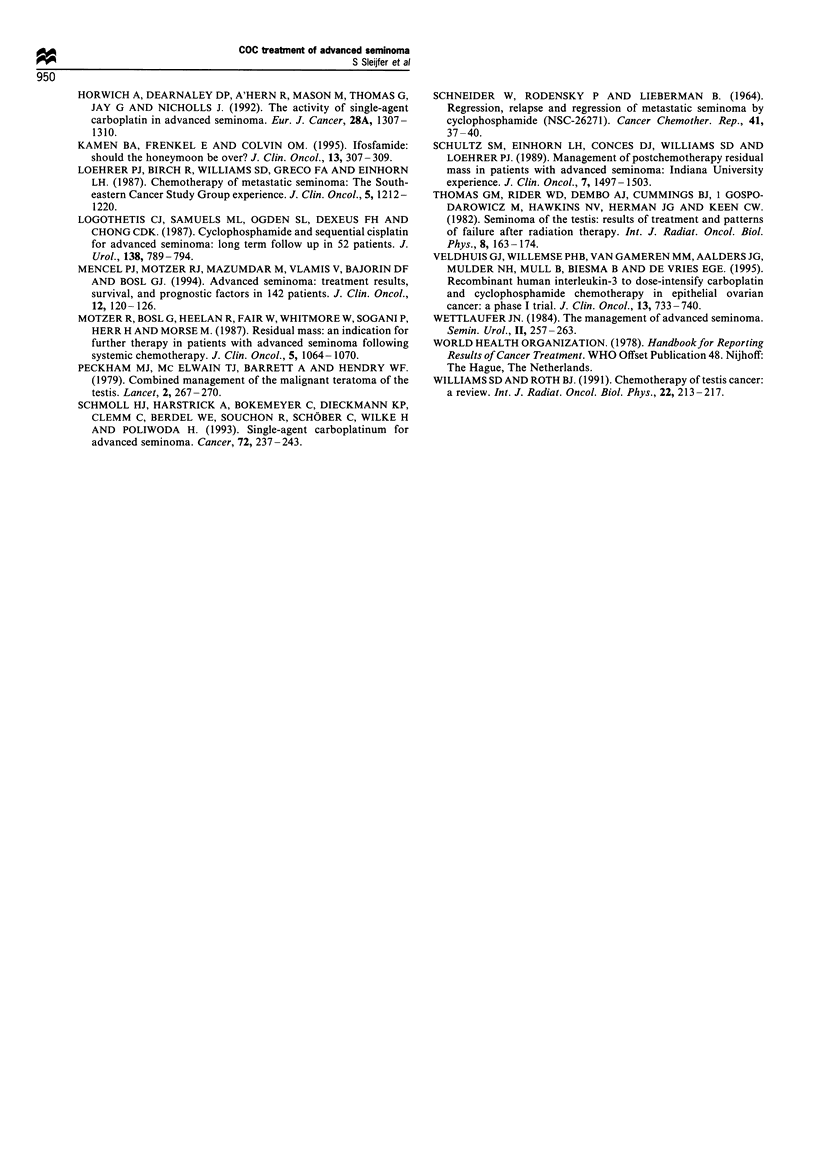# Treatment of advanced seminoma with cyclophosphamide, vincristine and carboplatin on an outpatient basis.

**DOI:** 10.1038/bjc.1996.462

**Published:** 1996-09

**Authors:** S. Sleijfer, P. H. Willemse, E. G. de Vries, W. T. van der Graaf, H. Schraffordt Koops, N. H. Mulder

**Affiliations:** Department of Internal Medicine, University Hospital, Groningen, The Netherlands.

## Abstract

This study describes the efficacy and toxicity of a combination regimen consisting of cyclophosphamide, vincristine (oncovin) and carboplatin (COC) for advanced seminoma on an outpatient basis. Twenty-seven patients (mean age 43 years, range 28-63 years) were classified as stage IIC (n = 5), stage IID (n = 12), stage III (n = 9) or stage IV (n = 1). Six had been treated with prior radiotherapy; elevated beta-HCG and elevated LDH serum levels were observed in 15 and 25 patients respectively. Patients were treated with four cycles of 750 mg m-2 cyclophosphamide intravenously (i.v.), 1.4 mg m-2 vincristine i.v. (maximum 2 mg) and carboplatin adjusted to creatinine clearance. Cycles were given at 3 week intervals. The median dose of carboplatin administered was 400 mg m-2 (range 300-450 mg m-2). Six patients [22%; 95% confidence interval (CI), 6-38%] achieved a complete response (CR), 19 (70%; 95% CI, 51-88%) a partial response and two (8%; 95% CI, 0 18%) showed only a response in tumour markers but not a reduction of retroperitoneal mass (NR). Post-chemotherapeutic masses were not removed surgically or irradiated. After a median follow-up of 26 months (range 5-69 months), two patients have died, one from cardiac arrest 2 years after achieving CR, the other with relapsed seminoma 5 months after therapy. None of the other patients relapsed. Main toxicity was haematological, with 22 patients (81%) experiencing thrombocytopenia WHO grade III/IV and 27 (100%) leucocytopenia WHO grade III/IV, requiring dose reduction in five patients. Seven patients experienced granulocytopenic fever. Non-haematological toxicity was rare. Peripheral neuropathy grade I was observed in four patients and grade III in one. Haemorrhagic cystitis occurred once. In conclusion, despite considerable haematological toxicity, COC is feasible on an outpatient basis, even after prior radiotherapy, and is an effective regimen for advanced seminoma with only 1/27 treatment failures after a median follow-up of 26 months.


British Journal of Cancer (1996) 74, 947-950
© 1996 Stockton Press All rights reserved 0007-0920/96 $12.00

### Treatment of advanced seminoma with cyclophosphamide, vincristine and carboplatin on an outpatient basis

S Sleijfer[1], PHB Willemse[1], EGE de Vries[1], WTA van der Graaf[1], H Schraffordt Koops[2] and NH Mulder[1]

[1]*Division of Medical Oncology of the Department of Internal Medicine and* [2]*Department of Surgical Oncology, University Hospital, Groningen, The Netherlands.*

**Summary** This study describes the efficacy and toxicity of a combination regimen consisting of cyclophosphamide, vincristine (oncovin) and carboplatin (COC) for advanced seminoma on an outpatient basis. Twenty-seven patients (mean age 43 years, range 28–63 years) were classified as stage IIC ($n=5$), stage IID ($n=12$), stage III ($n=9$) or stage IV ($n=1$). Six had been treated with prior radiotherapy; elevated $\beta$-HCG and elevated LDH serum levels were observed in 15 and 25 patients respectively. Patients were treated with four cycles of 750 mg m$^{-2}$ cyclophosphamide intravenously (i.v.), 1.4 mg m$^{-2}$ vincristine i.v. (maximum 2 mg) and carboplatin adjusted to creatinine clearance. Cycles were given at 3 week intervals. The median dose of carboplatin administered was 400 mg m$^{-2}$ (range 300–450 mg m$^{-2}$). Six patients [22%; 95% confidence interval (CI), 6–38%] achieved a complete response (CR), 19 (70%; 95% CI, 51–88%) a partial response and two (8%; 95% CI, 0–18%) showed only a response in tumour markers but not a reduction of retroperitoneal mass (NR). Post-chemotherapeutic masses were not removed surgically or irradiated. After a median follow-up of 26 months (range 5–69 months), two patients have died, one from cardiac arrest 2 years after achieving CR, the other with relapsed seminoma 5 months after therapy. None of the other patients relapsed. Main toxicity was haematological, with 22 patients (81%) experiencing thrombocytopenia WHO grade III/IV and 27 (100%) leucocytopenia WHO grade III/IV, requiring dose reduction in five patients. Seven patients experienced granulocytopenic fever. Non-haematological toxicity was rare. Peripheral neuropathy grade I was observed in four patients and grade III in one. Haemorrhagic cystitis occurred once. In conclusion, despite considerable haematological toxicity, COC is feasible on an outpatient basis, even after prior radiotherapy, and is an effective regimen for advanced seminoma with only 1/27 treatment failures after a median follow-up of 26 months.

**Keywords:** seminoma; vincristine; cyclophosphamide; carboplatin

Seminoma is a tumour that is highly sensitive to radiotherapy. Therefore, low-stage seminoma (Royal Marsden classification stage IIA/IIB) can be successfully treated with radiotherapy (Thomas *et al.*, 1982; Fosså *et al.*, 1989). Because of high relapse rates after radiotherapy in more advanced stages (Thomas *et al.*, 1982), such patients are commonly treated primarily with chemotherapy. Chemotherapeutic regimens used have been based on experiences obtained in the treatment of non-seminomas. Cisplatin-based combination chemotherapy has been shown to be very effective in the treatment of seminomas, and the combination consisting of etoposide and cisplatin with or without bleomycin can be considered as standard therapy nowadays (Mencel *et al.*, 1994; Williams *et al.*, 1991). Unfortunately, cisplatin-based regimens are characterised by toxic side-effects such as renal damage, neurotoxicity and ototoxicity and require hospitalisation. Carboplatin is a cisplatin analogue that lacks many of the side-effects of cisplatin and can be administered on an outpatient basis (Calvert *et al.*, 1985). Used as a single agent, carboplatin is active against seminoma (Schmoll *et al.*, 1993; Horwich *et al.*, 1989), but many patients relapse (Horwich *et al.*, 1992).

Cyclophosphamide (Logothesis *et al.*, 1987; Wettlaufer *et al.*, 1984) and vincristine (Fosså *et al.*, 1995; Wettlaufer *et al.*, 1984) are agents which have also been successfully used in combination with other drugs against testicular cancer.

This study describes the efficacy and toxicity of a combination regimen for advanced seminoma consisting of carboplatin, vincristine (oncovin) and cyclophosphamide (COC) administered on an outpatient basis.

Correspondence: NH Mulder, Division of Medical Oncology, Department of Internal Medicine, University Hospital, PO Box 30.001, 9700 RB Groningen, The Netherlands
Received 16 January 1996; revised 22 March 1996; accepted 17 April 1996

### Patients and methods

Between January 1989 and April 1995, 27 patients with histologically proven advanced seminoma ($\geqslant$ stage IIC) were entered in this study. All patients were thoroughly evaluated including measurement of $\beta$-human chorionic gonadotrophin ($\beta$-HCG), $\alpha$-fetoprotein ($\alpha$FP) and lactate dehydrogenase (LDH). Before treatment, patients were staged according to the Royal Marsden classification (Peckham *et al.*, 1979) by physical examination and computed tomography (CT) of abdomen and chest. Before and during each cycle patients were physically examined, accompanied by determination of haemoglobin, white blood count (WBC), platelets, creatinine clearance, liver function and tumour markers ($\beta$-HCG, $\alpha$FP and LDH).

Patients were treated with four cycles of COC given at 3-week intervals. Chemotherapy consisted of 750 mg m$^{-2}$ cyclophosphamide i.v., 1.4 mg m$^{-2}$ vincristine i.v. (maximum 2 mg) and carboplatin i.v. adjusted before each cycle to the creatinine clearance. At creatinine clearance below 100, between 100 and 120, between 120 and 140, or above 140 ml min$^{-1}$, carboplatin doses administered were 300, 350, 400 or 450 mg m$^{-2}$ respectively. The first cycle was given clinically, the other cycles were administered on an outpatient basis.

Within 4 weeks after administration of the last cycle, evaluation of response was performed by determination of tumour markers and CT scanning of previous abnormal lesions. Complete response (CR) was defined as complete disappearance of known sites of disease and normalisation of tumour markers. A more than 50% reduction of the sum of the products of the longest diameter and its perpendicular for all known measurable lesions was defined as partial response (PR). A no response (NR) was featured by a less than 50% reduction of the sum of the products of the longest diameter and its perpendicular.

After completion of chemotherapy, residual masses were not removed surgically nor irradiated and patients were followed by close observation. CT scanning during follow-up was only performed if indicated.

Toxicity was evaluated in all patients and scored according to the WHO criteria (WHO, 1978). Granulocytopenic fever was defined as temperature >38°C and WBC $<2\times10^9\ l^{-1}$. Postponement of a cycle was performed if the number of thrombocytes was below $75\times10^9\ l^{-1}$ just before administration. In case of neuropathy WHO grade III/IV vincristine was stopped. A 10% dose reduction of carboplatin was carried out if patients received transfusion of thrombocytes in the previous cycle.

To analyse differences between groups, $\chi^2$ was used. Only $P$-values < 0.05 were considered significant.

The study was approved by the medical ethics committee of the University Hospital Groningen. All patients gave informed consent.

### Results

### *Patient characteristics*

Patient characteristics at entry of the study are depicted in Table I. Two patients presented with an extragonadal seminoma, all other patients had a testicular seminoma. Six patients received prior radiotherapy on the abdomen for stage I or IIA–IIB seminoma (mean total doses 31 Gy, range 25–38 Gy) but relapsed after an initial response.

None of the patients had an elevated αFP serum level (normal < 5 μg $l^{-1}$). An elevated LDH serum level (normal < 235 IU $l^{-1}$) was observed in 25 patients. Mean value of the patients with an elevated LDH level was 928 IU $l^{-1}$ (range 242–3665). Fifteen patients had an elevated β-HCG serum level (normal < 2 μg $l^{-1}$) with a mean elevated level of 26.3 μg $l^{-1}$ (range 3–135).

### *Response to treatment*

Response to chemotherapy is shown in Table II. Six patients (22%; 95% confidence interval (CI), 6–38%) achieved a CR, 19 (70%; 95% CI, 51–88%) a PR and two patients (8%; 95% CI, 0–18%) showed NR. Of the 21 patients with a post-chemotherapeutic mass (19 PR and 2 NR), 11 patients had a residual mass larger than 3 cm.

After a median follow-up of 26 months (range 5–69 months), two patients have died. Two years after obtaining a CR, one patient, 44 years old, died from a cardiac arrest. This patient had slightly elevated cholesterol serum levels and a family history of cardiac disease. The other patient had a slightly elevated LDH serum level and an increasing residual mass 4 months after obtaining a NR. Complete surgical removal of the mass appeared to be impossible during laparotomy. Histology revealed necrosis and viable seminoma cells. Five months after completion of chemotherapy, this patient died from progressive disease. None of the other patients relapsed, so after a median follow-up of 26 months, 25 patients (93%; 95% CI, 83–100%) are alive and show no evidence of active disease. The median observation period in patients with a residual mass larger than 3 cm is 25 months (range 4–59 months).

**Table I** Patients' characteristics

| | |
|---|---|
| No. of patients | 27 |
| Mean age (years) (range) | 43 (28–63) |
| Previous radiotherapy | 6 |
| Stage | |
| IIC | 5 |
| IID | 12 |
| III | 9 |
| IV | 1 |
| Elevated αFP | 0 |
| Elevated β=HCG | 15 |
| Elevated LDH | 25 |

### *Toxicity of treatment*

All patients received four cycles, so in total 108 cycles were given. The median dose of carboplatin administered was 400 mg $m^{-2}$ (range 300–450 mg $m^{-2}$).

Because of haematological toxicity, a 10% dose reduction of carboplatin was carried out in five cycles (5%) in four patients. Vincristine was not administered in one patient in the last cycle because of neuropathy. Postponement of cycles was performed in 14 cycles (13%) in ten patients. Mean postponement was 4.5 days (range 2–7 days).

Toxicity is outlined in Table III. Main toxicity was

**Table II** Tumour responses and follow-up

| | | *Response* | | | *Follow-up* | |
|---|---|---|---|---|---|---|
| | *No.* | *CR* | *PR* | *NR* | *Relapsed* | *Deaths* |
| All patients | 27 | 6 | 19 | 2 | 1 | 2 |
| Previous radiotherapy | 6 | 5 | 1 | 0 | 0 | 0 |
| Stage | | | | | | |
| IIC | 5 | 1 | 4 | 0 | 0 | 0 |
| IID | 12 | 1 | 9 | 2 | 1 | 2 |
| III | 9 | 4 | 5 | 0 | 0 | 0 |
| IV | 1 | 0 | 1 | 0 | 0 | 0 |

**Table III** Toxicity

| | *No. of patients (%)* | *Cycles (%)* |
|---|---|---|
| Thrombocytopenia (WHO grade III/IV) | 22 (81) | 57 (53) |
| Thrombocyte transfusion | 11 (41) | 18 (17) |
| Leucocytopenia (WHO grade III/IV) | 27 (100) | 85 (79) |
| Granulocytopenic fever | 7 (26) | 8 (7) |
| Neuropathy (WHO grade III/IV) | 1 (4) | 1(1) |

haematological. Thrombocytopenia WHO grade III/IV was observed in 57 cycles (53%) in 22 patients requiring transfusion of thrombocytes in 18 cycles (17%) in 11 patients. No bleeding episodes occurred. Leucocytopenia grade III/IV was encountered in 85 cycles (79%) in 27 patients. Granulocytopenic fever occurred in eight cycles (7%) in seven patients. These patients were admitted and intensively treated with antibiotics. Two patients received granulocyte colony-stimulating factor (G-CSF). In patients receiving prior radiotherapy, occurrence of thrombocytopenia or leucocytopenia WHO grade III/IV was not different compared with the other patients.

Non-haematological toxicity was rare. Four patients experienced neuropathy grade I and one patient grade III. Haemorrhagic cystitis was observed in one patient.

### Discussion

Combination chemotherapy is the treatment of choice for patients presenting with advanced seminoma or relapsing after previous radiotherapy. The regimens used have been based on results obtained in the treatment of non-seminoma and consequently cisplatin-based regimens have become standard. Mencel *et al.* (1994) compared different platinum-based chemotherapeutic combinations and concluded that four cycles with etoposide and cisplatin is the most effective therapy leading to a response rate of approximately 95%. However, cisplatin-containing regimens are feared for their induction of auditory, neural or renal toxicity and usually require some form of hospitalisation.

Used at standard doses, carboplatin has several advantages over cisplatin with the most important feature being its lack of nephrotoxicity which makes hydration and hospitalisation unnecessary (Calvert *et al.*, 1985). Carboplatin used as a single agent has been shown to be active against seminoma resulting in a response level similar to that obtained with cisplatin-based combinations (Horwich *et al.* 1989, 1992; Schmoll *et al.*, 1993). However, Horwich *et al.* (1992) observed that 23% of the patients relapsed after a median follow-up of 3 years, a recurrence rate too high for this type of cancer. Therefore, we developed a carboplatin-containing multidrug regimen which would hopefully lead to a lower relapse rate.

Cyclophosphamide has been shown to be effective against seminoma as a single agent (Schneider *et al.*, 1964) and in combination with cisplatin (Logothetis *et al.*, 1987). Wettlaufer *et al.* (1984) were the first to use vincristine in the treatment of testicular cancer and treated seminoma patients with a combination of vincristine, cyclophosphamide and cisplatin followed by radiotherapy for residual lesions. In this study of 12 patients, these authors report 92% to be without evidence of disease after a median follow-up of 24 months. Recently, Fosså *et al.* (1995) modified Wettlaufer's regimen by substituting cyclophosphamide for ifosphamide yielding an effective regimen with a 3-year survival of 90%. This combination was however relatively toxic particularly with myelosuppression. Although remaining controversial, ifosphamide has been claimed to have several advantages over cyclophosphamide (Kamen *et al.*, 1995). Recently, Amato *et al.* (1995) treated advanced seminoma with a combination of carboplatin and cyclophosphamide resulting in 91% of the patients free of disease after a median follow-up of 35 months. However, compared with ifosphamide, cyclophosphamide has the merit of being suitable for administration on an outpatient basis.

In this study, we also modified the regimen as designed by Wettlaufer *et al.* (1984) by replacing cisplatin with carboplatin (COC). COC resulted in 93% of the patients free of disease after a median follow-up of 26 months, a figure equivalent to that achieved with other multidrug regimens (Fosså *et al.*, 1995; Gietema *et al.*, 1991; Loehrer *et al.*, 1987; Mencel *et al.*, 1994; Amato *et al.*, 1995).

Toxicity of COC was mainly myelosuppression with 100% of the patients experiencing leucocytopenia grade III/IV and 81% thrombocytopenia grade III/IV. This toxicity is comparable with studies using similar doses of carboplatin and cyclophosphamide for ovarian carcinoma (De Vries *et al.*, 1991; Biesma *et al.*, 1992). However, this kind of toxicity may be prevented partly by the addition of growth factors such as IL-3 (Biesma *et al.*, 1992; Veldhuis *et al.*, 1995) or GM-CSF (De Vries *et al.*, 1991).

Residual mass after chemotherapy for seminoma is a common phenomenon, but its management remains controversial. Several options have been suggested such as surgical removal (Motzer *et al.*, 1987), radiotherapy (Fosså *et al.*, 1987), biopsy to detect viable tumour cells or observation (Schultz *et al.*, 1989). Addition of surgery or radiotherapy, especially of large residual masses, would add to the overall toxicity of the treatment and therefore in this study patients were followed by close observation.

The only treatment failure in this study relapsed in a residual mass of 10 cm. Although large lesions have been described to be at increased risk, this risk does not seem to be high (1/11) after the regimen described here.

Shrinkage of post-chemotherapeutic mass is a phenomenon known to occur often in seminoma (Fosså *et al.*, 1995). However, we did not perform CT scans during follow-up, so in this study it is not possible to evaluate whether lesions observed directly after therapy disappeared in time.

Although the number of patients treated is small and the follow-up relatively short, it can be concluded that COC is an effective regimen for advanced seminoma resulting in a response level similar to that achieved by others. Besides that, its main toxicity, myelosuppression, may be partly prevented by the addition of growth factors, the advantage of COC over other regimens is its feasibility to treat on an outpatient basis.

### References

AMATO RJ, ELLERHORST J, BANKS M AND LOGOTHETIS CJ. (1995). Carboplatin and ifosfamide and selective consolidation in advanced seminoma. *Eur. J. Cancer*, **31A**, 2223–2228.

BIESMA B, WILLEMSE PHB, MULDER NH, SLEIJFER DTh, GIETEMA JA, MULL R, LIMBURG PC, BOUMA J, VELLENGA E AND DE VRIES EGE. (1992). Effects of interleukin-3 after chemotherapy for advanced ovarian cancer. *Blood*, **80**, 1141–1148.

CALVERT AH, HARLAND SJ, NEWELL DR, SIDIK ZH AND HARRAP KR. (1985). Phase I studies with carboplatin at the Royal Marsden Hospital. *Cancer. Treat. Rev.*, **12**, (suppl A), 51–57.

DE VRIES EGE, BIESMA B, WILLEMSE PHB, MULDER NH, STERN AC, AALDERS JG AND VELLENGA E. (1991). A double-blind placebo-controlled study with GM-CSF during chemotherapy for ovarian carcinoma. *Cancer Res.*, **51**, 116–122.

FOSSÅ SD, BORGE L, AASS N, JOHANNESSEN NB, STENWIG AE AND KAALHUS O. (1987). The treatment of advanced metastatic seminoma: experience in 55 cases. *J. Clin. Oncol.*, **5**, 1071–1077.

FOSSÅ SD, AASS N AND KAALHUS O. (1989). Radiotherapy for testicular seminoma stage I: treatment results and long-term post-irradiation morbidity in 365 patients. *Int. J. Radiat. Oncol. Biol. Phys.*, **16**, 383–388.

FOSSÅ SD, DROZ JP, STOTER G, KAYE SB, VERMEYLEN K, SYLVESTER R AND THE MEMBERS OF THE EORTC GU GROUP (1995). Cisplatin, vincristine, ifosphamide combination chemotherapy of metastatic seminoma: results of EORTC trial 30874. *Br. J. Cancer*, **71**, 619–624.

GIETEMA JA, WILLEMSE PHB, MULDER NH, OLDHOFF J, DE VRIES EGE AND SLEIJFER DTH. (1991). Alternating cycles of PVB and BEP in the treatment of patients with advanced seminoma. *Eur. J. Cancer*, **27**, 1376–1379.

HORWICH A, DEARNALEY DP, DUCHESNE GM, WILLIAMS M, BRADA M AND PECKHAM MJ. (1989). Simple nontoxic treatment of advanced metastatic seminoma with carboplatin. *J. Clin. Oncol.*, **7**, 1150–1156.

HORWICH A, DEARNALEY DP, A'HERN R, MASON M, THOMAS G, JAY G AND NICHOLLS J. (1992). The activity of single-agent carboplatin in advanced seminoma. *Eur. J. Cancer*, **28A**, 1307–1310.

KAMEN BA, FRENKEL E AND COLVIN OM. (1995). Ifosfamide: should the honeymoon be over? *J. Clin. Oncol.*, **13**, 307–309.

LOEHRER PJ, BIRCH R, WILLIAMS SD, GRECO FA AND EINHORN LH. (1987). Chemotherapy of metastatic seminoma: The Southeastern Cancer Study Group experience. *J. Clin. Oncol.*, **5**, 1212–1220.

LOGOTHETIS CJ, SAMUELS ML, OGDEN SL, DEXEUS FH AND CHONG CDK. (1987). Cyclophosphamide and sequential cisplatin for advanced seminoma: long term follow up in 52 patients. *J. Urol.*, **138**, 789–794.

MENCEL PJ, MOTZER RJ, MAZUMDAR M, VLAMIS V, BAJORIN DF AND BOSL GJ. (1994). Advanced seminoma: treatment results, survival, and prognostic factors in 142 patients. *J. Clin. Oncol.*, **12**, 120–126.

MOTZER R, BOSL G, HEELAN R, FAIR W, WHITMORE W, SOGANI P, HERR H AND MORSE M. (1987). Residual mass: an indication for further therapy in patients with advanced seminoma following systemic chemotherapy. *J. Clin. Oncol.*, **5**, 1064–1070.

PECKHAM MJ, MC ELWAIN TJ, BARRETT A AND HENDRY WF. (1979). Combined management of the malignant teratoma of the testis. *Lancet*, **2**, 267–270.

SCHMOLL HJ, HARSTRICK A, BOKEMEYER C, DIECKMANN KP, CLEMM C, BERDEL WE, SOUCHON R, SCHÖBER C, WILKE H AND POLIWODA H. (1993). Single-agent carboplatinum for advanced seminoma. *Cancer*, **72**, 237–243.

SCHNEIDER W, RODENSKY P AND LIEBERMAN B. (1964). Regression, relapse and regression of metastatic seminoma by cyclophosphamide (NSC-26271). *Cancer Chemother. Rep.*, **41**, 37–40.

SCHULTZ SM, EINHORN LH, CONCES DJ, WILLIAMS SD AND LOEHRER PJ. (1989). Management of postchemotherapy residual mass in patients with advanced seminoma: Indiana University experience. *J. Clin. Oncol.*, **7**, 1497–1503.

THOMAS GM, RIDER WD, DEMBO AJ, CUMMINGS BJ, 1 GOSPODAROWICZ M, HAWKINS NV, HERMAN JG AND KEEN CW. (1982). Seminoma of the testis: results of treatment and patterns of failure after radiation therapy. *Int. J. Radiat. Oncol. Biol. Phys.*, **8**, 163–174.

VELDHUIS GJ, WILLEMSE PHB, VAN GAMEREN MM, AALDERS JG, MULDER NH, MULL B, BIESMA B AND DE VRIES EGE. (1995). Recombinant human interleukin-3 to dose-intensify carboplatin and cyclophosphamide chemotherapy in epithelial ovarian cancer: a phase I trial. *J. Clin. Oncol.*, **13**, 733–740.

WETTLAUFER JN. (1984). The management of advanced seminoma. *Semin. Urol.*, **II**, 257–263.

WORLD HEALTH ORGANIZATION. (1978). *Handbook for Reporting Results of Cancer Treatment*. WHO Offset Publication 48. Nijhoff: The Hague, The Netherlands.

WILLIAMS SD AND ROTH BJ. (1991). Chemotherapy of testis cancer: a review. *Int. J. Radiat. Oncol. Biol. Phys.*, **22**, 213–217.